# Standardization and reference ranges for whole blood platelet function measurements using a flow cytometric platelet activation test

**DOI:** 10.1371/journal.pone.0192079

**Published:** 2018-02-01

**Authors:** Dana Huskens, Yaqiu Sang, Joke Konings, Lisa van der Vorm, Bas de Laat, Hilde Kelchtermans, Mark Roest

**Affiliations:** 1 Cardiovascular Research Institute Maastricht, Maastricht University Medical Centre, Maastricht, the Netherlands; 2 Synapse Research Institute, Maastricht, the Netherlands; 3 Department of Clinical Chemistry and Hematology, Gelre Hospitals, Apeldoorn, The Netherlands; Royal College of Surgeons in Ireland, IRELAND

## Abstract

**Introduction:**

Platelet function testing with flow cytometry has additional value to existing platelet function testing for diagnosing bleeding disorders, monitoring anti-platelet therapy, transfusion medicine and prediction of thrombosis. The major challenge is to use this technique as a diagnostic test. The aim of this study is to standardize preparation, optimization and validation of the test kit and to determine reference values in a population of 129 healthy individuals.

**Methods:**

Platelet function tests with 3 agonists and antibodies against P-selectin, activated αIIbβ3 and glycoprotein Ib (GPIb), were prepared and stored at -20°C until used. Diluted whole blood was added and platelet activation was quantified by the density of activation markers, using flow cytometry. Anti-mouse Ig κ particles were included to validate stability of the test and to standardize results. Reference intervals were determined.

**Results:**

Blood stored at room temperature (RT) for up to 4h after blood donation and preheated/tested at 37°C resulted in stable results (%CV<10%), in contrast to measuring at RT. The intra-assay %CV was <5%. Incubation of anti-mouse Ig κ particles with antibodies stored for up to 12 months proved to give a stable fluorescence. The inter-individual variation measured in the 129 individuals varied between 23% and 37% for P-selectin expression and αIIbβ3 activation, respectively.

**Conclusions:**

The current study contributes to the translation of flow cytometry based platelet function testing from a scientific tool to a diagnostic test. Platelet function measurements, using prepared and stored platelet activation kits, are reproducible if executed at 37°C. The reference ranges can be validated in clinical laboratories and ongoing studies are investigating if reduced platelet reactivity in patients with bleeding complications can be detected.

## Introduction

The assessment of platelet (dys)function is of crucial relevance in several clinical settings, including: monitoring the response to antiplatelet treatment, evaluation of perioperative haemostasis, transfusion medicine and identification of bleeding disorders [1].

The current gold standard of testing platelet function for the diagnosis of bleeding disorders is light transmission aggregometry (LTA) [2]. However, LTA is laborious, relatively insensitive to small changes in platelet function and the outcome is affected by haemolysis and an abnormal platelet count [3]. Additionally, the test is poorly standardized, consumes large blood volumes and requires skilled technicians in specialized laboratories [4, 5].

Platelet function testing by flow cytometry in whole blood was first described by Shattil et al. [6]. Under current guidelines, the use of flow cytometry is recommended as an integral component of the investigation of platelet function disorders [7, 8]. In mild bleeding disorders, flow cytometric platelet activation shows a good correlation with LTA [9]. In acute coronary syndromes and after coronary stenting, expression of platelet activation markers predicts major adverse cardiac events and, in patients with atrial fibrillation, increased expression is a risk factor for silent cerebral infarction [5, 10, 11].

Flow cytometric analysis of platelet function in whole blood is a technique that has many advantages. First of all, flow cytometry measures within limited time the specific characteristics of a large number (e.g. 5000 or 10,000) of individual platelets. Furthermore, platelets are analysed in their physiological milieu of whole blood, including erythrocytes and leukocytes, both of which affect platelet activation [12, 13], without any pretreatment of the blood. This allows for measurement of both the baseline activation state of circulating platelets and their reactivity in response to various agonists without the influence of artefactual *in vitro* activation by preconditioning of blood [6]. Also, this method permits the detection of two specific activation-dependent modifications in the platelet surface membrane. The amount of activated αIIbβ3 receptors and the expression of P-selectin on the cell membrane correspond to the aggregation potential and granule release capacity of platelets, respectively [14–16]. The test has been used for measuring platelet function in bleeding disorders, during surgery, in platelet concentrates, and in patients with thrombocytopenia [17, 18]. Because only minimal volumes (5 μl) of blood are required, the technique is also suitable for neonatal studies [19, 20]. Unfortunately, there are many different protocols circulating and flow cytometry detection of platelet function is still only applied in research settings, due to the absence of standardisation between labs and instruments. In the past, the tests for the detection of platelet function by flow cytometry required fresh preparation of reaction mixes. This was not only laborious but also detrimental for the reproducibility of the test. A simultaneous improvement of reproducibility and of the workload was achieved by preparing large batches of stable reaction mixes, that are aliquoted in small reaction tubes and stored in a freezer for several months. Another issue in flow cytometry-based platelet function testing is the temperature of the blood before and during the test. Currently, the majority of studies using this technique are performed at room temperature (RT) [21, 22], while it is known that RT by itself is a co-trigger of platelet activation [23, 24]. Finally, no consensus is reached on the optimal time frame post blood drawing in which the test should be performed to exclude *ex vivo* platelet activation. Some studies claim that blood samples should be processed within 30 minutes [6], while others advice to wait for a stable response, achieved 1 to 2 hours post blood collection [25].

The aims of this study are (1) to solve the discussion about the optimal temperature for measuring flow cytometric platelet function, (2) to standardize, optimize and validate the preparation of the test kits and (3) to determine reference values in a healthy population.

## Materials and methods

### Reagents

Platelet agonists used for measuring platelet function include the P2Y_12_ agonists ADP (01897, Sigma-Aldrich, the Netherlands), the protease activated receptor (PAR)-1 agonist thrombin receptor activator peptide (TRAP-6 (SFLLRN), H-2936; Bachem, Germany) and the glycoprotein VI (GPVI) agonist collagen-related peptide (CRP, a generous gift by Professor Farndale, university of Cambridge, UK). Professor Farndale identified fragments of collagen, synthesized and assembled in active, triple-helical conformation and in this study, a single batch of cross-linked CRP (CRP-XL) was used. The antibodies used in this test were FITC-conjugated monoclonal antibody PAC1 directed against the activated αIIbβ3 receptor, PE-conjugated monoclonal antibody anti-P-selectin (CD62P, clone AK4) and APC-conjugated anti-CD42b (GPIb) monoclonal antibody (clone HIP1), all purchased from BD Pharmingen (NJ, USA). Anti-mouse Ig κ particles and negative control particles were also purchased from BD Pharmingen.

### Preparation of the platelet activation tests

Optimal agonist concentrations were determined in 5 healthy donors of which the data are shown (S1 Fig). Test strips consisting of the four optimal test conditions (no agonist, 30 μmol/L TRAP, 5 μg/ml CRP and 30 μmol/L ADP) were prepared in advance and stored at -20°C. Each reaction mixture with a total volume of 20 μl consists of 2 μl FITC-conjugated PAC1, 1.5 μl PE-conjugated anti-P-selectin and 0.5 μl APC-conjugated anti-CD42b with or without agonist in HEPES-buffered saline (HBS, 10 mmol/L HEPES, 150 mmol/L NaCl, 1 mmol/L MgSO_4_, 5 mmol/L KCL, pH 7.4).

#### Preparation of the reaction mixtures for the beads

In each platelet activation test, 3 additional conditions to detect binding of antibodies to anti-mouse Ig κ particles or negative control particles were included. The negative control condition (NC) contains 2 μl FITC-conjugated PAC1 and 1.5 μl PE-conjugated anti-P-selectin in HEPES-buffered saline (total volume of 20 μl). Two positive control (PC) conditions were prepared: PC1 contains 2 μl FITC-conjugated PAC1 and PC2 contains 1.5 μl PE-conjugated anti-P-selectin in HEPES-buffered saline (total volume of 20 μl).

### Performing the platelet activation tests

Test strips were thawed and shortly centrifuged. All tests were performed at 37°C, unless stated otherwise. Whole blood was stored at RT for at least 30 min and maximum 4h, incubated at 37°C for 10 minutes and diluted 1:4 in pre-heated HEPES-buffered saline to minimize the formation of platelet aggregates. From this diluted blood, 5 μl was added to each reaction mixture (20 μl, final dilution 1:20) and the tests were incubated for exactly 20 minutes at 37°C. At the same time, 5 μl of negative control particles and 5 μl of anti-mouse Ig κ particles were added to the NC condition or to both PC1 and PC2, respectively.

Reactions were stopped by adding 250 μl fixation solution (137 mmol/L NaCl, 2.7 mmol/L KCl, 1.12 mmol/L NaH_2_PO_4_, 1.15 mmol/L KH_2_PO_4_, 10.2 mmol/L Na_2_HPO_4_, 4 mmol/L EDTA, 0.5% formaldehyde). Upon fixation, platelet activation markers were stable from day 2 to day 8 (change in MFI <5%, S2 Fig), but most samples were analysed one day after the experiment (day 2).

Flow cytometry was used to discriminate platelets from other cells, using the forward and sideward scatter pattern and by gating on the CD42b positive cells. Fluorescent intensity in the FITC gate and PE gate was selected to determine activated αIIbβ3 and P-selectin density, respectively, and results are expressed as median fluorescent intensity (MFI).

### Study population

Our study protocol was evaluated by the local medical ethical board (Medical Ethical Committee of Maastricht University Medical Center). The study population consisted of 129 healthy adult individuals, aged 20–65 years. All participants did not take any oral anticoagulant or anti-platelet drugs for at least two weeks, did not have a history of thrombosis or bleeding and gave full written informed consent according to the Helsinki declaration. Experiments were conducted at the Synapse Research Institute in accordance with approved guidelines and regulations. Blood was collected aseptically by antecubital puncture via a 21-gauge needle (0.8x32 mm) into vacuum tubes (1 volume trisodium citrate 0.105M to 9 volumes blood) (BD Vacutainer System/Greiner). The blood was kept at RT and used within 4 hours from withdrawal. Cell counts in whole blood were performed with a Coulter Counter analyzer (Beckman Coulter, Woerden, The Netherlands).

### Statistics

Beads were used to normalize the flow cytometric data with the following equation:

$$100\times \left({\text{MFI}}_{\text{a}}-{\text{MFI}}_{\text{c}}\right)/\left({\text{MFI}}_{\text{PC}}-{\text{MFI}}_{\text{NC}}\right)$$

Where MFI_a_ was the MFI of the activated platelets, MFI_c_ the MFI of the unstimulated platelets, MFI_PC_ the MFI measured on the positive control beads and MFI_NC_ the MFI of the negative control beads.

For the reference values, outlier analysis was performed using the D/R method (Dixon). One outlier was detected and excluded (P-selectin expression in response to ADP measured on the Accuri as well as the Canto). Normality was tested using the Shapiro Wilk test and except for αIIbβ3 receptor activation in response to CRP, data were not normal distributed. Continuous variables were expressed as median and interquartile range (25%-75%). Also, mean and standard deviation (SD) were shown and inter-individual variability (%CV) was calculated for each variable as 100 x (SD/mean). Reference intervals were obtained using non-parametric calculation methods, in accordance with the recommendation in the latest CLSI guidelines [26]. More precisely, the lower reference interval limit was estimated as the 2.5^th^ percentile, and the upper limit as the 97.5^th^ percentile of the distribution. Groups were compared using the Mann-Whitney U test for independent samples.

## Results

### Optimisation of the platelet function test

#### Determination of the optimal temperature conditions

To study the effects of the storage temperature on platelet function, blood was drawn and stored at 37°C or RT. After 30 minutes, whole blood was diluted and added to reaction mixtures containing the agonists TRAP, CRP or ADP, or a control buffer. In the control condition without agonist, no αIIbβ3 receptor activation or P-selectin expression was detectable, regardless of the storage temperature (S3 Fig). Incubation of whole blood with platelet agonists resulted in a strong increase in the amount of αIIbβ3 receptor activation and in the expression of P-selectin. Activation of the αIIbβ3 receptor in response to TRAP, CRP or ADP as well as P-selectin expression in response to ADP was significantly lower at 37°C compared to RT. On the other hand, the temperature did not significantly affect the P-selectin expression in response to TRAP or CRP.

**Fig 1:**
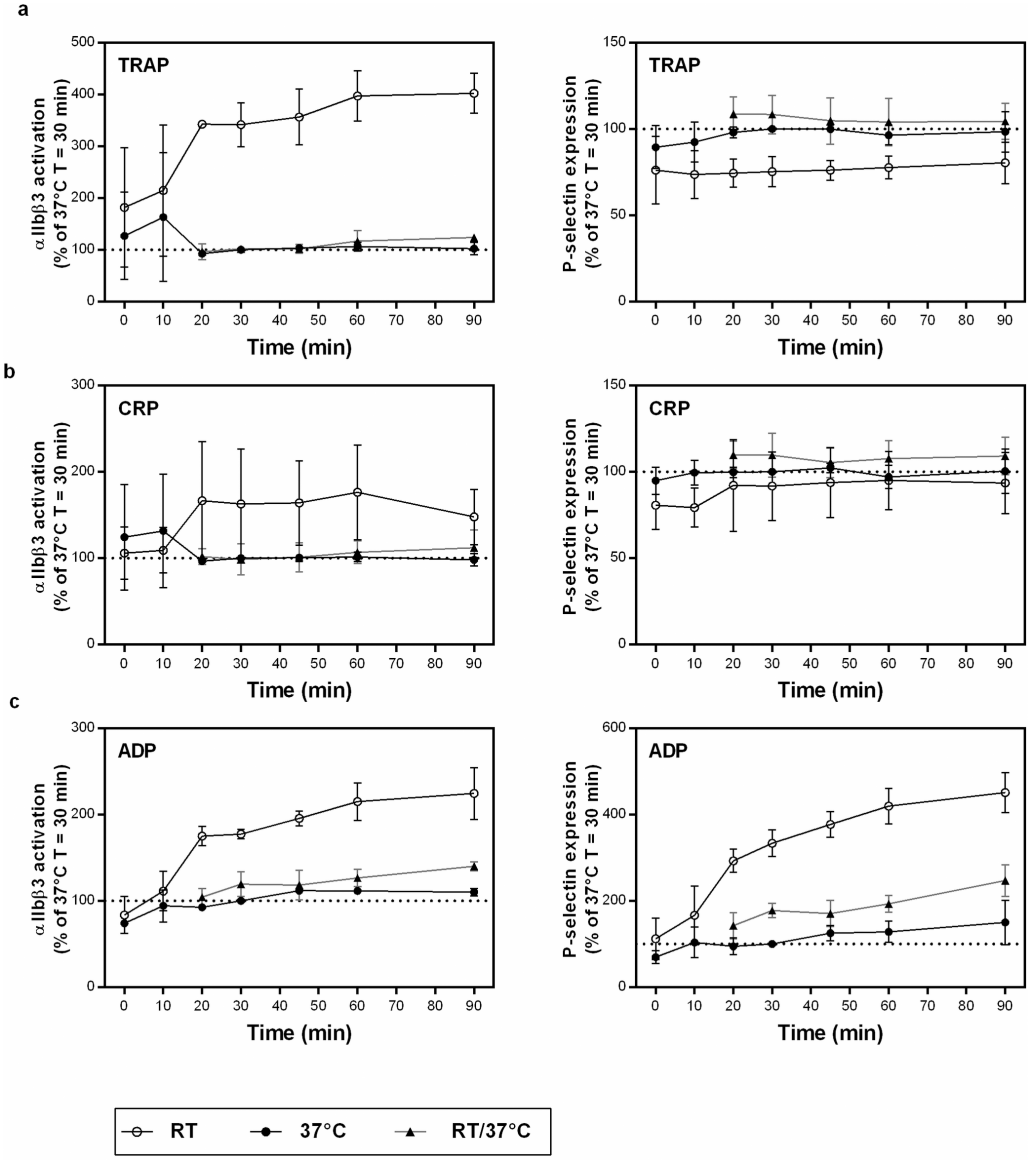
Effect of temperature on platelet function testing by flow cytometry. Immediately after blood donation, blood was stored at RT or at 37°C. At each time point (X-axis), platelet activation in response to TRAP (panel a), CRP (panel b) or ADP (panel c) was tested. For the 37°C condition, blood was immediately placed at 37° and platelet function tests were performed at 37°C. For the RT condition, blood was kept at RT and the tests were performed at RT. For the RT/37°C condition, blood was kept at RT and 10 min before performing the platelet function test, blood was placed at 37°C and tests were performed at 37°C. The median fluorescence intensity (MFI) of αIIbβ3 receptor activation (left panels) and P-selectin expression (right panels) was recorded and expressed as the percentage compared to the MFI of the 37°C condition measured after 30 min at 37°C. Data are presented as mean ± SD (n = 3 to 5).

When measuring at RT, standardization of the time from blood collection to the start of platelet function testing has been advised to reduce the variation in results [27]. We investigated this in more detail and blood was drawn from 3–5 donors and stored at 37°C or RT. At different time points (immediately (t = 0) and after 10 min, 20 min, 30 min, 45 min, 60 min and 90 min), blood was diluted and added to the platelet function reaction mixtures. In clinical setting, constant incubation at 37°C is challenging because the collected blood needs to be transported to the laboratory. Therefore, we included a RT/37°C condition, in which the blood stored at RT (from 10 min up till 80 min) was incubated at 37°C for 10 min before the platelet activation test was initiated. Subsequently, reactions were incubated for 20 min at 37°C or at RT followed by fixation of the cells. The MFI for αIIbβ3 receptor activation and P-selectin expression were recorded and the percentages compared to the 37°C condition measured 30 minutes after blood donation are shown in Fig 1. Pre-analytical storage of blood at RT showed a time-dependent increase in platelet reactivity. Blood samples that were stored pre-analytically for up to 20 min at RT after blood collection had a significant lower αIIbβ3 receptor activation in response to TRAP, CRP or ADP than blood that was pre-analytically stored at RT for longer periods (Fig 1). The same pattern was observed for P-selectin expression if platelets were activated with ADP. In contrast to RT, pre-analytical storage of blood at 37°C hardly influenced platelet reactivity from 20 min up to 90 min after blood collection. Interestingly, the responses of platelets stored at RT but preheated for 10 min at 37°C before performing the platelet function test were comparable to those of platelets stored at 37°C immediately after blood collection (Fig 1). These findings indicate that blood should be preheated to 37°C, at least 10 min before platelet function analysis.

Additionally, the variability introduced by pre-analytical storage of the blood up to 4 hours at RT was tested in 4 donors (Table 1). Prior to the experiment, blood was stored for at least 30 minutes and maximally 4 hours at RT, followed by a 10 minutes incubation at 37°C. Acceptable variability was found when platelets were activated with TRAP and CRP (coefficient of variation less than 10%; Table 1), while platelet activation by ADP showed more variability due to pre-analytical storage of blood. Given the enormous practical advantage of blood storage at RT without the need to standardize the time between blood collection and test initiation, we selected the RT/37°C condition for further experiments.

**Table 1:** Assay variation when blood is stored at RT up till 240 min followed by 10 min preincubation at 37°C prior to performing the platelet function test.

		Variability 30 min—240 min (%CV)				
		Donor 1	Donor 2	Donor 3	Donor 4	MEAN %CV
αIIbβ3 activation	TRAP	9.3	7.9	7.3	6.3	**7.7**
	CRP	3.1	6.4	2.5	7.2	**4.8**
	ADP	18.3	5.5	7.3	10.6	**10.4**
P-selectin expression	TRAP	5.8	6.1	10.1	5.5	**6.9**
	CRP	6.2	6.2	8.4	7.0	**7.0**
	ADP	24.4	7.9	24.2	16.8	**18.3**

Blood was stored for 30, 60, 120, 180 and 240 min before it was preincubated for 10 min at 37°C.

#### Stability of platelet function tests stored at -20°C

**Fig 2:**
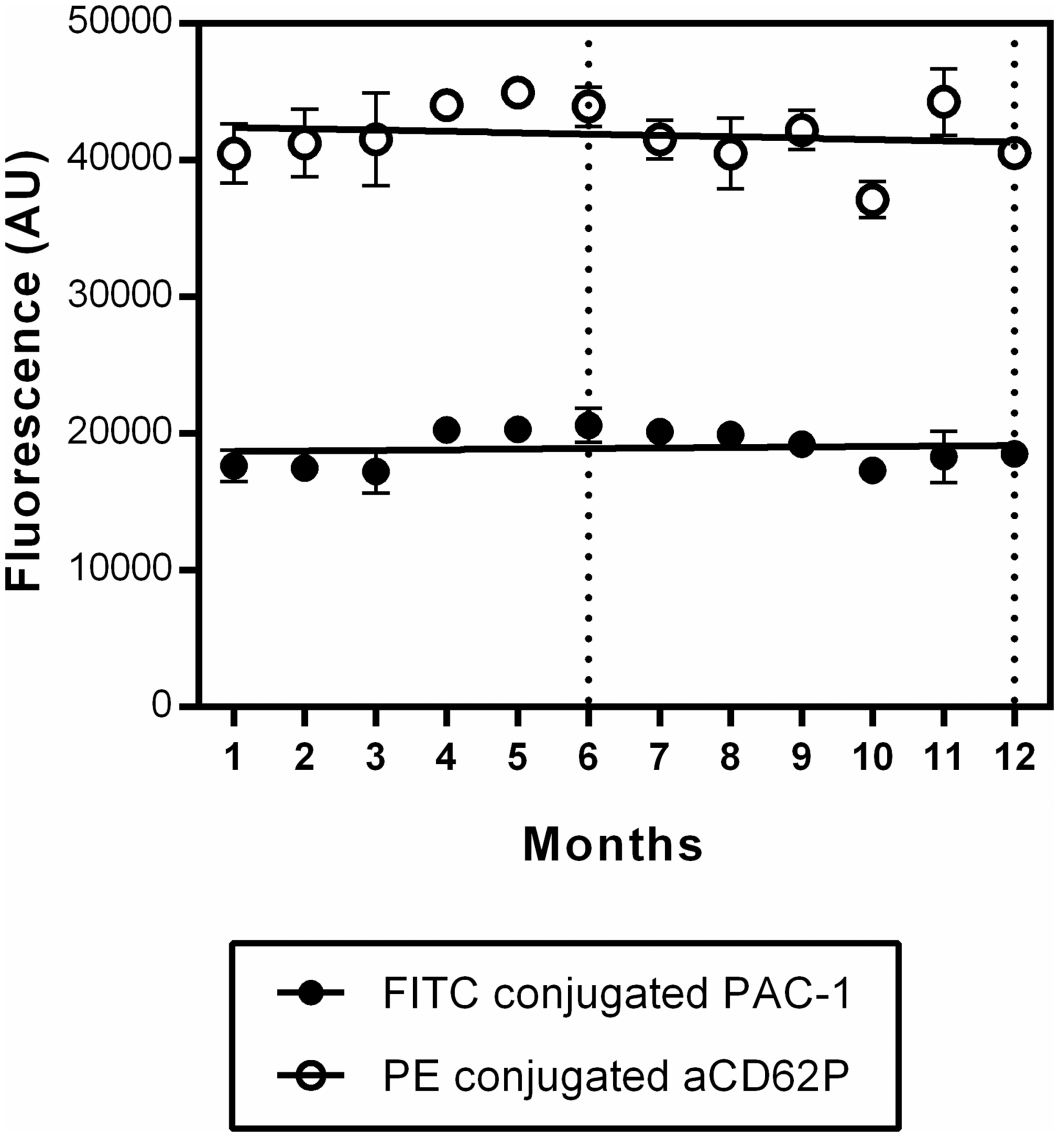
Determination of the stability of the antibodies stored at -20°C. Tests consisting of two positive control solutions (containing either the PAC-1 antibody (PC1) or the anti-P-selectin antibody (PC2)) and one negative control (containing both the PAC-1 and the anti-P-selectin antibodies), were stored at -20°C for up to 12 months. Every month, four tests of each condition were thawed. Anti-mouse Ig κ particles or negative control beads were added accordingly and incubated for 20 min at 37°C. Binding of the FITC-conjugated PAC-1 as well as the PE-conjugated anti-P-selectin was recorded as MFI measured on the PC beads minus MFI of the NC beads. After 6 months and 12 months a new batch of beads was used (indicated by the dashed lines). Data are presented as mean ± SD (n = 4) and linear regression lines are shown. Every month, anti-mouse Ig κ particles or negative control beads were added to four tests accordingly.

In this platelet function assay, reaction mixtures containing the complete package of conjugated antibodies and agonists, were stored at -20°C. To test the stability of the antibodies present in the platelet function tests, we included anti-mouse Ig κ particles, which bind the FITC conjugated PAC-1 as well as the PE conjugated anti-P-selectin antibodies in two separate conditions (PC1 and PC2). The beads, with approximately the same size as platelets, provided distinct positive stained populations which can also be used to determine the compensation levels of the flow cytometer. Tests consisting of two positive control solutions and one negative control, were stored for up to 12 months. Every month, anti-mouse Ig κ particles or negative control beads were added to four tests accordingly. Binding of the FITC-conjugated PAC-1 antibody as well as the PE-conjugated anti-P-selectin antibody was recorded as MFI measured on the PC beads minus MFI of the NC beads (Fig 2). After 6 months and 12 months a new batch of beads was used. The binding of the anti-P-selectin antibody to the PC beads resulted in a higher fluorescence than the binding of the PAC-1 antibody. Importantly, fluorescence measured was constant in time for both antibodies (slope of the linear regression line not significantly different from zero with P-values of 0.5 and 0.4 for PAC-1 and anti-P-selectin, respectively).

#### Determination of the precision of the platelet activation test

To obtain intra-assay precision data of our test, we performed replicate experiments (n = 10) in 4 healthy donors. For each donor, these experiments were carried out in parallel after preheating the blood for 10 minutes at 37°C, using the same batch of platelet function tests. Platelets were activated with TRAP, CRP and ADP and incubated with FITC-conjugated PAC-1 and PE-conjugated anti-P-selectin, and MFI were measured to determine αIIbβ3 receptor activation and P-selectin expression on the platelet membrane, respectively. At the same time, the binding of these antibodies to positive control beads was measured. For each donor, the mean values with the SD and %CV of the MFI are depicted in Table 2. For each condition, the mean intra-assay variation was found to be below 5% with the exception of the platelet activation in response to ADP.

**Table 2:** Intra assay variability of the platelet function test and beads.

	Intra-assay variability								
	Donor 1 (n = 10)		Donor 2 (n = 10)		Donor 3 (n = 10)		Donor 4 (n = 10)		MEAN %CV
	Mean±SD	%CV	Mean±SD	%CV	Mean±SD	%CV	Mean±SD	%CV	
*αIIbβ3 activation*									
TRAP	1050±38	3.6	1738±71	4.1	972±51	5.2	1196±84	7.0	**5.0**
CRP	8475±100	1.2	10384±568	5.5	7162±195	2.7	8297±374	4.5	**3.5**
ADP	8077±984	12.2	6243±280	4.5	4351±144	3.3	3912±161	4.1	**6.0**
*P-selectin expression*									
TRAP	8804±340	3.9	9621±232	2.4	7965±377	4.7	7603±363	4.8	**3.9**
CRP	8995±227	2.5	10205±244	2.4	8246±382	4.6	8308±190	2.3	**3.0**
ADP	5219±1560	29.9	1048±155	14.8	923±272	29.4	599±46	7.7	**20.4**
*Positive control beads*									
PAC-1-FITC	18209±134	0.7	21047±573	2.7	19795±468	2.4	19584±331	1.7	**1.9**
aCD62P-PE	39510±692	1.8	46287±1833	4.0	43513±732	1.7	40992±1361	3.3	**2.7**

Due to the need of fresh blood, a true inter-assay CV determined on consecutive days could not be assessed.

#### Usage of beads to reduce the lot-to-lot variability and comparison of data obtained by different flow cytometers

In a next set of experiments, we investigated if normalisation of the results of the platelet activation test by results obtained on beads reduces variability between lots of tests and/or allows the comparison of data obtained by different flow cytometers.

**Fig 3:**
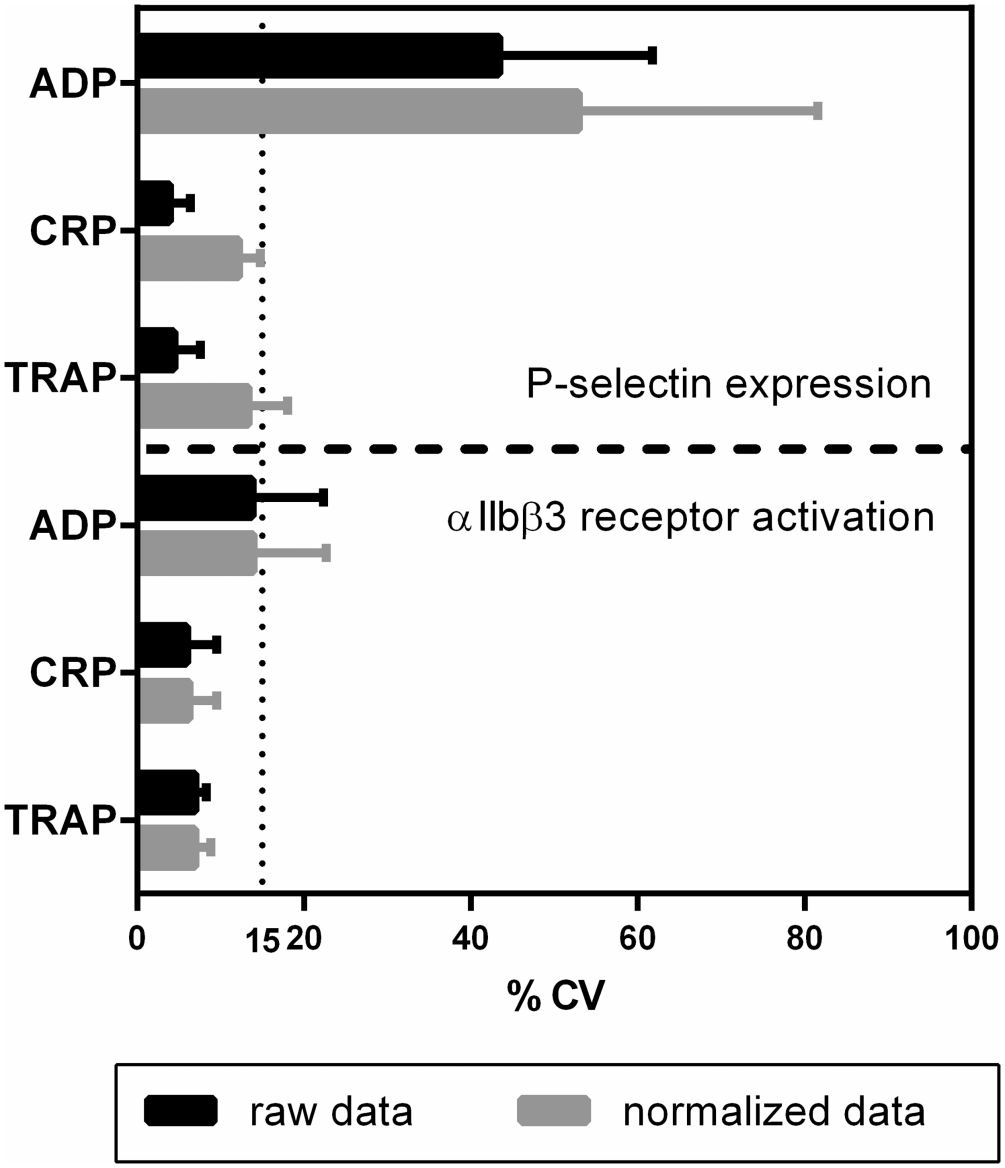
Influence of normalization on the lot-to-lot variability of the platelet activity test. Five lots of platelet function tests were prepared and stored at -20°C for 1, 4, 7, 8 and 9 months. Whole blood of 4 donors was used to study the variability between the lots. For each condition MFI values were recorded and normalized using the fluorescence measured on the NC and PC beads. Coefficient of variations for both the raw data and normalized data are shown with their corresponding SD.

The lot-to-lot variability was determined by measuring platelet activation in 4 donors using 5 different test lots per donor. The tests, prepared and stored at -20°C for 1, 4, 7, 8 or 9 months, consisted of a control condition without agonist, three conditions with agonists and three conditions for NC and PC beads (Materials and methods). Data were normalized as described in materials and methods. For each donor, the lot-to-lot variability was determined by calculating the %CV of the raw data as well as the normalized data for each condition (mean %CVs are shown in Fig 3). Normalization did not affect the lot-to-lot variability for αIIbβ3 receptor activation, however, the lot-to-lot variability did increase for P-selectin when data were normalized (Fig 3). With the exception of P-selectin expression in response to ADP, the lot-to-lot variability for all conditions was below 15%.

Measuring the same sample on different flow cytometers often results in different absolute MFI values due to different lasers and fluorescent detectors in the systems. To investigate if normalization of the data allows the comparison of results obtained with different flow cytometers, blood was collected from 126 donors and the platelet function assay was performed. The tests were analysed on a BD Accuri and a BD FACSCanto, and for each donor, the MFI values as well as the normalized values of the two cytometers were compared. To create a clear general picture, values of the first 50 donors are shown in the S4 Fig. Mean MFI values measured on the FACSCanto/Accuri were 509/1105 AU, 2909/6398 AU and 1661/3733 AU for αIIbβ3 receptor activation and 5141/5102 AU, 5169/5206 AU and 421/323 AU for P-selectin expression in response to TRAP, CRP and ADP, respectively. Normalization of the MFI values resulted in mean normalized values measured on FACSCanto/Accuri (%CV) of 7.3/6.8 (7%), 41.9/39.4 (6%) and 23.9/22.9 (6%) for αIIbβ3 receptor activation and 18.0/12.6 (26%), 18.1/12.8 (25%) and 1.5/0.8 (48%) for P-selectin expression in response to TRAP, CRP and ADP, respectively.

Additionally, platelet function tests of 2 donors were analysed on an Accuri and a Navios flow cytometer of Beckman Coulter. Mean MFI values were measured on Navios/Accuri and were 3.9/739.5, 31.1/6039.3, 13.5/2543 for αIIbβ3 receptor activation and 22.2/8320.5, 22.6/8618.8 and 1.6/477.5 for P-selectin expression in response to TRAP, CRP and ADP, respectively. Mean normalized values measured on Navios/Accuri (%CV) of 3.9/3.7 (2%), 31.3/30.5 (3%) and 13.5/12.7 (4%) for αIIbβ3 receptor activation and 13.2/16.3 (15%), 13.4/16.9 (17%) and 0.9/0.9 (6%) for P-selectin expression in response to TRAP, CRP and ADP, respectively.

### Measuring platelet function in a healthy population

#### Determination of inter-individual variability and reference intervals of platelet function

Demographic data of 129 healthy individuals (63 females and 66 males, median 32.0 years) together with blood counts are summarized in Table 3. Concerning the blood counts, the haemoglobin levels, haematocrit and red blood cell counts were significantly increased and platelet counts significantly reduced in male compared to female samples.

**Table 3:** Baseline data of 129 healthy individuals.

	All	Women (n = 63)	Men (n = 66)	P-values
Age, years	32 (27–44)	32 (27–44)	31 (26–44)	ns
Haemoglobin, mmol/L	9.0 (8.3–9.6)	8.36 (8.0–8.9)	9.5 (9.1–9.8)	<0.0001
Haematocrit, %	42 (39–44)	39 (38–42)	44 (42–45)	<0.0001
Red blood cell count, *10^12^/L	4.9 (4.5–5.1)	4.6 (4.4–4.9)	5.0 (4.8–5.2)	<0.0001
Platelet count, *10^9^/L	316 (273–370)	332 (289–391)	295 (264–359)	0.004
White blood cell count, *10^9^/L	5.7 (5.0–6.7)	5.9 (5.1–7.0)	5.4 (4.8–6.5)	ns

Medians and interquartile ranges (25%-75%) are indicated.

The inter-individual variability (%CV) as well as reference intervals (2.5 percentile- 97.5 percentile) for P-selectin expression and αIIbβ3 receptor activation in response to the agonists TRAP, CRP and ADP were determined in these 129 healthy volunteers (Table 4). Samples were measured on an Accuri flow cytometer and a FACSCanto. For CRP, two relatives with a previously unknown GPVI deficiency were excluded. Apart from the reference values, medians, interquartile ranges (IQRs), minimum and maximum values of each test parameter are indicated. Looking at the inter-individual variation, for TRAP and CRP the %CV for P-selectin expression and αIIbβ3 receptor activation proved to be acceptable and in the range of 23 to 37%.

**Table 4:** Inter-individual variation and reference intervals of the platelet function test.

	N	Mean (% of beads)	Stdev	%CV	Median	IQR (25%-75%)	Reference Interval (2.5%-97.5%)	Min	Max
**Accuri**									
*αIIbβ3 activation*									
TRAP	129	6.8	2.5	36.5	6.5	5.1–8.2	2.3–12.8	2.2	13.2
CRP	127	40.0	9.8	24.4	40.7	34.0–47.3	18.1–60.6	10.7	65.4
ADP	129	22.8	8.7	38.0	22.6	16.9–27.0	7.7–43.8	4.6	45.4
*P-selectin expression*									
TRAP	129	12.7	4.0	31.0	12.5	9.5–14.9	6.6–22.4	5.4	26.0
CRP	127	13.1	4.1	31.3	12.5	9.9–15.4	7.2–23.8	6.4	26.2
ADP	128	0.8	0.7	86.4	0.6	0.2–1.1	0.09–2.5	0.1	3.7
**Canto**									
*αIIbβ3 activation*									
TRAP	126	7.3	2.6	35.9	7.1	5.4–9.0	2.7–13.8	2.4	14.5
CRP	124	42.4	9.7	22.9	41.9	36.3–48.7	20.9–63.1	11.0	68.8
ADP	126	23.9	8.8	36.8	23.3	17.3–28.4	8.5–45.0	5.9	46.6
*P-selectin expression*									
TRAP	126	18.0	4.5	24.7	17.8	14.4–20.5	10.8–29.4	10.0	34.1
CRP	124	18.3	4.4	24.3	17.6	14.9–20.8	11.7–28.1	9.0	34.7
ADP	125	1.4	1.1	81.3	1.1	0.6–1.8	0.3–5.0	0.1	5.4

#### Effect of sex and age on platelet activation

The effect of sex on platelet activation was investigated for all agonists. P-selectin expression and αIIbβ3 receptor activation in response to ADP were significantly higher in females compared to males (S5 Fig). The same trend was observed for the agonist TRAP and CRP, although the differences did not reach statistical significance.

Looking at the effect of age of the healthy volunteers on platelet activation, weak but significant correlations were found for αIIbβ3 receptor activation in response to TRAP and ADP (Spearman correlation coefficient r = 0.19 (p = 0.03), r = 0.22 (p = 0.01), respectively) and for P-selectin expression in response to ADP (r = 0.18 (p = 0.04)).

## Discussion

This study contributes to the development of an *in vitro* diagnostic test that measures platelet activation by flow cytometry. We investigated the effect of pre-analytical temperature and storage conditions on platelet function and showed that blood can be stored at RT after blood collection if the blood is preheated at 37°C, 10 min before platelet function is measured at 37°C. Results obtained by three types of flow cytometers (BD Accuri, BD FACS Canto, Beckman Navios) were found to be not comparable, unless the tests are calibrated by calibrator beads. Another important qualification of diagnostic tests is the reproducibility, which is acceptable for our test as shown by the low %CV (with the exception of P-selectin expression in response to ADP). Finally, we have determined platelet function in 129 healthy volunteers to obtain the normal ranges, inter-subject variation and to study the effects of age and sex on platelet function.

Our goal was to develop a user-friendly, reliable and quick platelet function test using three platelet agonists (TRAP, CRP and ADP) and two platelet activation markers (αIIbβ3 receptor activation and P-selectin expression). This reveals a limitation of our study because adding additional agonists (PAR4-selective agonist, arachidonic acid, ristocetin) and activation markers (CD40L, CD63, annexin-V, mepacrine) would result in a more detailed picture on platelet activation, however, this was beyond the scope of this study.

Currently, important discrepancies exist in the published flow cytometric platelet function protocols. Despite the evidence of spontaneous platelet activation at RT, most tests are performed at RT and no consensus is reached on the ideal time interval between blood collection and initiation of the test [21, 22, 25, 27–30]. As shown by our results, platelet function at RT is affected by storage time between blood collection and analysis, while pre-analytical storage of blood at 37°C will not affect platelet function over time, if the time frame is from 20 min up to 4 hours after blood collection. Interestingly, the effect of low temperature on platelet function was reversible, as platelets stored at RT and subsequently incubated for 10 minutes at 37°C were equally responsive as platelets stored continuously at 37°C (irrespective of the time after blood donation). Taking into account its advantages in a clinical setting (allowing blood transport at RT from the clinics to the laboratory), the RT/37°C condition was preferred for further experiments.

Incubation at RT resulted in higher signals for αIIbβ3 activation in response to TRAP, CRP and ADP and P-selectin expression in response to ADP in comparison with incubation at 37°C. This is in agreement with previous studies reporting that *in vitro* hypothermia enhances platelet αIIbβ3 activation and P-selectin expression in response to platelet agonists [23, 24, 27]. This also resulted in a higher optimal concentration of agonist (determined by the shoulder of the dose-effect curve) when measuring at 37°C, in comparison with other studies testing platelet activation at RT [25, 31, 32]. Further studies on patient populations will determine if this increase in agonist concentration influences the sensitivity of the test. The difference in MFI (RT versus 37°C) regarding αIIbβ3 activation seems to be more distinct for TRAP than for the other agonists, likely reflecting a fundamental difference in their mechanisms of platelet activation. Of note, the temperature-dependent binding kinetics of the PAC-1 antibody contributes to the higher signals for αIIbβ3 activation at RT [33]. In our view, measuring a stable platelet function response over time is more important than achieving extremely high fluorescent signals.

It has been suggested that flow cytometry-based platelet function measurements are laborious. This impression is based on old school test set-ups, which rely on freshly prepared reaction mixes and depend on skilled staff to ensure reproducible results. In this report, we show that reaction mixtures (containing both agonists and antibodies) can be stored stably at -20°C for at least one year. Using anti-mouse Ig κ particles to capture the antibodies of a reaction mixture, a strong stable fluorescent signal was detected. The stability of the agonists in the reaction mixtures stored at -20°C is also shown by the seasonal variation study started in February 2016 in which no consecutive decline of platelet function was measured over time. The advantage of the stable storage of reaction mixtures is that the test becomes less time consuming and more user friendly. Additionally, one batch of tests can be prepared per clinical study to avoid lot-to-lot variability.

Another issue in platelet function testing is the standardisation within and between laboratories. The current study describes a new approach, which uses anti-mouse Ig κ particles. We observed comparable αIIbβ3 activation (FITC signal) between different flow cytometers while the raw data differed enormously. In contrast, P-selectin expression (PE-signal) measured on different flow cytometers was more complex to calibrate. The much higher fluorescent signal measured on the positive control beads for the PE-conjugated anti-P-selectin may provide an explanation for this discrepancy. This reasoning may also explain why the usage of beads increases the lot to lot variability for P-selection expression. In the future, other beads with a lower amount of antibody binding sites will be developed to deal with this problem.

For the identification of patients with platelet dysfunction, it is essential to establish appropriate reference intervals. We have determined reference values for platelet function by flow cytometry in more than 120 individuals, as recommended by the latest CLSI guidelines [26].

In our healthy population, platelet activation in response to ADP was higher in women than in men and for this parameter, gender-specific reference values can be found in S5 Fig. Previously, it was demonstrated that platelets of women were significantly more active [34, 35] due to the conversion of a greater proportion of αIIbβ3 receptors into the active confirmation after agonist stimulation [36]. In contrast, a number of small studies failed to detect any differences in the reactivity of platelets between men and women [37–40]. Since we aimed to determine reference values in a broad normal population presenting at the clinic, we did not aim to determine gender-specific reference ranges. Furthermore, we found a low but significant correlation between platelet function and age. This is in agreement with a recent review on the effects of ageing on platelet function concluding that platelet function increases during middle age [41].

The reference intervals will serve as guidance to identify patients at risk for bleeding and thrombosis. We were able to reduce pre-analytical and analytical variables that affect platelet function. However, the measurement variation between different flow cytometers may still have an influence on platelet function results. Therefore, we emphasize that each laboratory should validate these reference intervals using their specific experimental conditions. Validation is typically performed in 20 normal individuals, and if no more than two results are outside our proposed reference intervals a laboratory may adopt our proposed reference intervals [26].

Studies investigating normal ranges for platelet function in healthy donors have shown an inter-individual variation of 16%-32%, especially at low and medium doses of agonists, with a considerable overlap in responses between individuals with and without reported bleeding problems [42–44]. These data correspond with our data showing an inter-individual variability of around 30% (apart from P-selectin expression in response to ADP).

Comparing the different agonists in our study, in most individuals ADP induces a low expression of P-selectin on the platelet outer membrane. This can be explained by the fact that ADP is a weak agonist for granule release. P-selectin expression is therefore in the noise range, which results in poor reproducibility. This in contrast with a strong agonist, such as thrombin or collagen, that can trigger granule secretion even when aggregation is prevented [45]. Therefore, we do not recommend to use P-selectin expression in response to ADP as a biomarker to predict bleeding, but the test may be useful in the prediction of thrombotic events.

## Conclusions

We optimized the flow cytometric platelet function assay by determining pre-analytical conditions and by preparing ready to use kits including calibrator beads. This resulted in stable user-friendly platelet function tests with acceptable intra-assay variability. Furthermore, this is the first study determining reference intervals in a large healthy cohort. These reference intervals can be validated in clinical laboratories and ongoing studies are investigating if we can detect reduced platelet activation in patients with bleeding complications.

## Supporting information

S1 Fig Determination optimal agonist concentration. Whole blood of 5 donors was incubated for 10 min at 37°C and subsequently, platelets were activated for 20 min at 37°C with TRAP (panel A), CRP (panel B) and ADP (panel C). Dose-dependent activation curves were created for both αIIbβ3 receptor activation (left panels) and P-selectin expression (right panels). The dotted line represents the concentration of the agonists used in the subsequent experiments.
(DOCX)

S2 Fig Stability of the platelet activation markers after fixation. At day 1, blood from 1 donor was added to two reaction mixtures consisting of an antibody mixture with the agonist CRP. After incubation for 20 minutes at 37°C, samples were fixated and analysed on the flow cytometer on different days. The change in median fluorescence intensity (MFI) over time is shown. The change was calculated as percentage of the MFI at day 2 (n = 2).
(DOCX)

S3 Fig Effect of temperature on platelet function testing by flow cytometry. Immediately after blood collection, blood was stored at 37°C (panel A) or RT (panel B) for 30 minutes. Platelet activation was tested in a control condition (grey) and after activation with TRAP (blue), CRP (green) or ADP (red) for 20 minutes at 37°C (panel A) or RT (panel B). The fluorescence histograms of αIIbβ3 receptor activation (left panels) and P-selectin expression (right panels) of one representative experiment are shown.
(DOCX)

S4 Fig Normalisation of data to compare results measured on different flow cytometers. Samples of 126 donors were measured on an Accuri and on a FACSCanto flow cytometer. Both αIIbβ3 receptor activation (panels A-B) and P-selectin expression (panels C-D) of the first 50 donors are presented as MFI values (panels A and C) and normalized data (panels B and D).
(DOCX)

S5 Fig Effect of sex on platelet activation. Both αIIbβ3 receptor activation and P-selectin expression in response to TRAP, CRP and ADP was determined in blood of 129 healthy volunteers. Normalized data were calculated for males (n = 66) and females (n = 63). Median and IQR are indicated. The grey areas delineated by the dotted lines represent the reference intervals of the total population (2.5 percentile– 97.5 percentile). Because platelet activation in response to ADP was significantly higher in females, reference intervals for males (blue) and females (red) were indicated. ** p < 0.01; *** p < 0.001 using the Mann-Whitney u test.
(DOCX)

S1 Database Database. All raw data underlying Figs 1–3, Tables 1–4 and S1, S2, S4 and S5 Figs are included in the database.
(DOCX)
