# Supplementary material for: Standardization and reference ranges for whole blood platelet function measurements using a flow cytometric platelet activation test
Source: PLoS One. 2018 Feb 1;13(2):e0192079. doi: 10.1371/journal.pone.0192079 (PMC5794146; doi:10.1371/journal.pone.0192079)
Supplement: S1 Fig — Whole blood of 5 donors was incubated for 10 min at 37°C and subsequently, platelets were activated for 20 min at 37°C with TRAP (panel A), CRP (panel B) and ADP (panel C). Dose-dependent activation curves were created for both αIIbβ3 receptor activation (left panels) and P-selectin expression (right panels). The dotted line represents the concentration of the agonists used in the subsequent experiments. (DOCX) [file pone.0192079.s001.docx]

**S1 Fig Determination optimal agonist concentration.** Whole blood of 5 donors was incubated for 10 min at 37°C and subsequently, platelets were activated for 20 min at 37°C with TRAP (panel A), CRP (panel B) and ADP (panel C). Dose-dependent activation curves were created for both αIIbβ3 receptor activation (left panels) and P-selectin expression (right panels). The dotted line represents the concentration of the agonists used in the subsequent experiments.
