# Supplementary material for: Standardization and reference ranges for whole blood platelet function measurements using a flow cytometric platelet activation test
Source: PLoS One. 2018 Feb 1;13(2):e0192079. doi: 10.1371/journal.pone.0192079 (PMC5794146; doi:10.1371/journal.pone.0192079)
Supplement: S2 Fig — At day 1, blood from 1 donor was added to two reaction mixtures consisting of an antibody mixture with the agonist CRP. After incubation for 20 minutes at 37°C, samples were fixated and analysed on the flow cytometer on different days. The change in median fluorescence intensity (MFI) over time is shown. The change was calculated as percentage of the MFI at day 2 (n = 2). (DOCX) [file pone.0192079.s002.docx]

**S2 Fig Stability of the platelet activation markers after fixation.** At day 1, blood from 1 donor was added to two reaction mixtures consisting of an antibody mixture with the agonist CRP. After incubation for 20 minutes at 37°C, samples were fixated and analysed on the flow cytometer on different days. The change in median fluorescence intensity (MFI) over time is shown. The change was calculated as percentage of the MFI at day 2 (n=2).
