# Supplementary material for: Standardization and reference ranges for whole blood platelet function measurements using a flow cytometric platelet activation test
Source: PLoS One. 2018 Feb 1;13(2):e0192079. doi: 10.1371/journal.pone.0192079 (PMC5794146; doi:10.1371/journal.pone.0192079)
Supplement: S3 Fig — Immediately after blood collection, blood was stored at 37°C (panel A) or RT (panel B) for 30 minutes. Platelet activation was tested in a control condition (grey) and after activation with TRAP (blue), CRP (green) or ADP (red) for 20 minutes at 37°C (panel A) or RT (panel B). The fluorescence histograms of αIIbβ3 receptor activation (left panels) and P-selectin expression (right panels) of one representative experiment are shown. (DOCX) [file pone.0192079.s003.docx]

**S3 Fig Effect of temperature on platelet function testing by flow cytometry.** Immediately after blood collection, blood was stored at 37°C (panel A) or RT (panel B) for 30 minutes. Platelet activation was tested in a control condition (grey) and after activation with TRAP (blue), CRP (green) or ADP (red) for 20 minutes at 37°C (panel A) or RT (panel B). The fluorescence histograms of αIIbβ3 receptor activation (left panels) and P-selectin expression (right panels) of one representative experiment are shown.


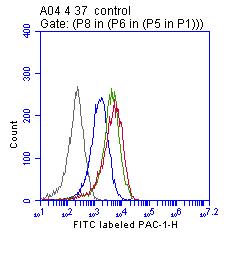

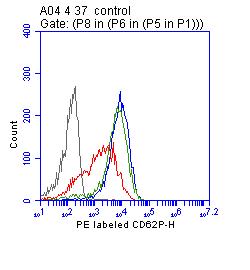

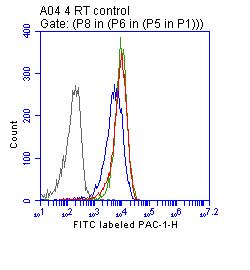

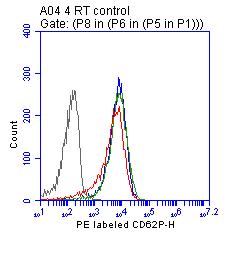


**A**

**B**

Expression activated αIIbβ3 receptor

Expression activated αIIbβ3 receptor

P-selectin expression

P-selectin expression

198

1492

4064

4835

121

8374

6464

1453

154

5337

9325

8886

198

7139

7370

4959
