# Supplementary material for: Standardization and reference ranges for whole blood platelet function measurements using a flow cytometric platelet activation test
Source: PLoS One. 2018 Feb 1;13(2):e0192079. doi: 10.1371/journal.pone.0192079 (PMC5794146; doi:10.1371/journal.pone.0192079)
Supplement: S4 Fig — Samples of 126 donors were measured on an Accuri and on a FACSCanto flow cytometer. Both αIIbβ3 receptor activation (panels A-B) and P-selectin expression (panels C-D) of the first 50 donors are presented as MFI values (panels A and C) and normalized data (panels B and D). (DOCX) [file pone.0192079.s004.docx]

**S4 Fig Normalisation of data to compare results measured on different flow cytometers.** Samples of 126 donors were measured on an Accuri and on a FACSCanto flow cytometer. Both αIIbβ3 receptor activation (panels A-B) and P-selectin expression (panels C-D) of the first 50 donors are presented as MFI values (panels A and C) and normalized data (panels B and D).
