# Supplementary material for: Standardization and reference ranges for whole blood platelet function measurements using a flow cytometric platelet activation test
Source: PLoS One. 2018 Feb 1;13(2):e0192079. doi: 10.1371/journal.pone.0192079 (PMC5794146; doi:10.1371/journal.pone.0192079)
Supplement: S5 Fig — Both αIIbβ3 receptor activation and P-selectin expression in response to TRAP, CRP and ADP was determined in blood of 129 healthy volunteers. Normalized data were calculated for males (n = 66) and females (n = 63). Median and IQR are indicated. The grey areas delineated by the dotted lines represent the reference intervals of the total population (2.5 percentile– 97.5 percentile). Because platelet activation in response to ADP was significantly higher in females, reference intervals for males (blue) and females (red) were indicated. ** p < 0.01; *** p < 0.001 using the Mann-Whitney u test. (DOCX) [file pone.0192079.s005.docx]

**S5 Fig Effect of sex on platelet activation.** Both αIIbβ3 receptor activation and P-selectin expression in response to TRAP, CRP and ADP was determined in blood of 129 healthy volunteers. Normalized data were calculated for males (n=66) and females (n=63). Median and IQR are indicated. The grey areas delineated by the dotted lines represent the reference intervals of the total population (2.5 percentile – 97.5 percentile). Because platelet activation in response to ADP was significantly higher in females, reference intervals for males (blue) and females (red) were indicated. ** p < 0.01; *** p < 0.001 using the Mann-Whitney u test

**Platelet function reference intervals in response to ADP**

|  | **Reference intervals (2.5%-97.5%)** | |
| --- | --- | --- |
|  | ***αIIbβ3 activation*** | ***P-selectin expression*** |
| **General** | 7.7-43.8 | 0.09-3.7 |
| **Males** | 7.4-41.9 | 0.07-2.6 |
| **Females** | 7.2-44.0 | 0.1-3.0 |
