# Supplementary material for: Standardization and reference ranges for whole blood platelet function measurements using a flow cytometric platelet activation test
Source: PLoS One. 2018 Feb 1;13(2):e0192079. doi: 10.1371/journal.pone.0192079 (PMC5794146; doi:10.1371/journal.pone.0192079)
Supplement: S1 Database — All raw data underlying Figs 1–3, Tables 1–4 and S1, S2, S4 and S5 Figs are included in the database. (DOCX) [file pone.0192079.s006.docx]

**Supporting information**

**S1 Determination optimal agonist concentration.** Whole blood of 5 donors was incubated for 10 min at 37°C and subsequently, platelets were activated for 20 min at 37°C with TRAP (panel A), CRP (panel B) and ADP (panel C). Dose-dependent activation curves were created for both αIIbβ3 receptor activation (left panels) and P-selectin expression (right panels). The dotted line represents the concentration of the agonists used in the subsequent experiments.

**S2** **Stability of the platelet activation markers after fixation.** At day 1, blood from 1 donor was added to two reaction mixtures consisting of an antibody mixture with the agonist CRP. After incubation for 20 minutes at 37°C, samples were fixated and analysed on the flow cytometer on different days. The change in median fluorescence intensity (MFI) over time is shown. The change was calculated as percentage of the MFI at day 2 (n=2).

**S3 Effect of temperature on platelet function testing by flow cytometry.** Immediately after blood collection, blood was stored at 37°C (panel A) or RT (panel B) for 30 minutes. Platelet activation was tested in a control condition (grey) and after activation with TRAP (blue), CRP (green) or ADP (red) for 20 minutes at 37°C (panel A) or RT (panel B). The fluorescence histograms of αIIbβ3 receptor activation (left panels) and P-selectin expression (right panels) of one representative experiment are shown.


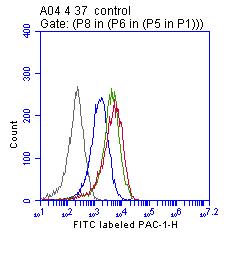

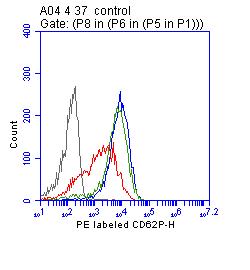

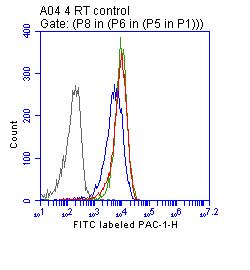

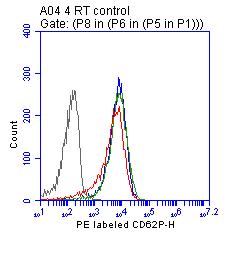


**A**

**B**

Expression activated αIIbβ3 receptor

Expression activated αIIbβ3 receptor

P-selectin expression

P-selectin expression

198

1492

4064

4835

121

8374

6464

1453

154

5337

9325

8886

198

7139

7370

4959

**S4 Normalisation of data to compare results measured on different flow cytometers.** Samples of 126 donors were measured on an Accuri and on a FACSCanto flow cytometer. Both αIIbβ3 receptor activation (panels A-B) and P-selectin expression (panels C-D) of the first 50 donors are presented as MFI values (panels A and C) and normalized data (panels B and D).

**S5 Effect of sex on platelet activation.** Both αIIbβ3 receptor activation and P-selectin expression in response to TRAP, CRP and ADP was determined in blood of 129 healthy volunteers. Normalized data were calculated for males (n=66) and females (n=63). Median and IQR are indicated. The grey areas delineated by the dotted lines represent the reference intervals of the total population (2.5 percentile – 97.5 percentile). Because platelet activation in response to ADP was significantly higher in females, reference intervals for males (blue) and females (red) were indicated. ** p < 0.01; *** p < 0.001 using the Mann-Whitney u test

**Platelet function reference intervals in response to ADP**

|  | **Reference intervals (2.5%-97.5%)** | |
| --- | --- | --- |
|  | ***αIIbβ3 activation*** | ***P-selectin expression*** |
| **General** | 7.7-43.8 | 0.09-3.7 |
| **Males** | 7.4-41.9 | 0.07-2.6 |
| **Females** | 7.2-44.0 | 0.1-3.0 |
